# Efficacy of Compound Betamethasone Periocular Injection Combined With Oral Mycophenolate Mofetil in Active Thyroid‐Associated Ophthalmopathy: A Pilot Study

**DOI:** 10.1155/ije/9591206

**Published:** 2025-12-31

**Authors:** Chunchun Xiao, Yiwei Liu, Wan Zhou, Yan Xing, Shandong Ye, Bin Huang

**Affiliations:** ^1^ Department of Endocrinology and Metabolism, The First Affiliated Hospital of USTC, University of Science and Technology of China, Hefei, China, ustc.edu.cn; ^2^ West China Hospital, Sichuan University, Chengdu, China, scu.edu.cn

**Keywords:** compound betamethasone, mycophenolate mofetil, periocular injection, thyroid-associated ophthalmopathy

## Abstract

**Objective:**

To evaluate the efficacy of combined therapy of compound betamethasone periocular injection and oral mycophenolate mofetil (MMF) in treating active thyroid‐associated ophthalmopathy (TAO).

**Methods:**

A retrospective study was conducted on 17 patients with active TAO treated with compound betamethasone periocular injection combined with oral MMF from February 2022 to August 2023, involving a total of 31 eyes.

**Results:**

In one eye, the Clinical Activity Score (CAS) decreased by less than two points, while in the remaining eyes, the CAS decreased by two points or more. Patients exhibited decreased proptosis, a reduction in average extraocular muscle thickness, and improvement in diplopia in six cases. Subgroup analysis indicated that this regimen showed no significant difference in efficacy for patients treated with radioactive iodine therapy or oral methimazole medication at baseline.

**Conclusion:**

Combined therapy of compound betamethasone periocular injection and oral MMF is an effective approach for treating active TAO.

## 1. Introduction

Thyroid‐associated ophthalmopathy (TAO) is an autoimmune inflammatory disorder primarily involving the orbital tissues, most commonly in patients with Graves’ disease but occasionally in those with euthyroidism or hypothyroidism [1, 2]. Its pathogenesis remains incompletely understood, with autoimmune, genetic, and environmental factors—such as smoking, infection, and radioactive iodine exposure—playing contributory roles [3, 4]. Clinically, TAO manifests as eyelid retraction, exophthalmos, periorbital edema, impaired ocular motility, and in severe cases, optic nerve compression, which can severely affect patients’ appearance, quality of life, and psychological well‐being.

Glucocorticoids remain the first‐line therapy during the active phase of TAO and can be administered systemically or locally. Although systemic corticosteroids are effective, their use is limited by significant adverse effects and contraindications in some patients. Periocular injection of triamcinolone acetonide (TA) has been reported to alleviate inflammation and improve ocular symptoms; however, TA often causes local irritation, crystallization, and tissue adhesion, making repeated injections difficult and potentially leading to increased intraocular pressure (IOP). Compound betamethasone, composed of 2‐mg betamethasone sodium phosphate and 5‐mg betamethasone dipropionate, offers both rapid and sustained anti‐inflammatory effects with minimal local irritation, making it suitable for repeated periocular administration [5].

Mycophenolate mofetil (MMF), a selective inhibitor of inosine monophosphate dehydrogenase (IMPDH), suppresses B‐ and T‐lymphocyte activation and exhibits both immunosuppressive and anti‐inflammatory properties [6, 7]. Several studies have shown that combining corticosteroids with MMF yields superior efficacy and safety compared with corticosteroid monotherapy [8–10]. The 2021 European Group on Graves’ Orbitopathy (EUGOGO) guidelines also recommend low‐dose MMF in combination with intravenous methylprednisolone for moderate‐to‐severe active TAO [11]. Based on these findings, this study retrospectively analyzed 17 patients (31 eyes) with active TAO (CAS ≥ 3) treated with periocular compound betamethasone injections combined with oral MMF, aiming to evaluate the clinical efficacy and safety of this regimen as a potential alternative to systemic corticosteroid therapy.

## 2. Study Participants and Methods

### 2.1. Study Population

A retrospective collection of patients visiting the Endocrinology Department of the First Affiliated Hospital of the University of Science and Technology of China (Anhui Provincial Hospital) from February 2022 to August 2023 was conducted. A total of 21 patients underwent superficial orbital injections of compound betamethasone, with 17 patients (comprising 31 eyes) included in the study. Inclusion criteria: patients with a Clinical Activity Score (CAS) of three or higher in the affected eye(s) (CAS includes spontaneous retrobulbar pain, pain on eye movement, eyelid erythema, eyelid edema, conjunctival erythema, conjunctival edema, and swelling of the lacrimal caruncle, each scored as one point).

Exclusion criteria: (1) patients who received oral or intravenous corticosteroid therapy within 6 weeks before superficial orbital injection of compound betamethasone. (2) Patients lacking pre‐ and posttreatment CASs, IOP measurements, and orbital CT data. (3) Patients who did not receive consecutive injections of compound betamethasone. All individuals provided informed consent to participate in this study, and approval was granted by the Medical Research Ethics Committee of the First Affiliated Hospital of University of Science and Technology of China (Anhui Provincial Hospital), with the ethics registration number: 2023‐RE‐325.

### 2.2. Study Method

Included patients received superficial orbital injections of compound betamethasone (Depo‐Medrone, 1 mL:5 mg:2 mg; Schering‐Plough Labo N.V., Belgium) administered every 2 weeks at a dosage of 7 mg per injection. Patients also received oral MMF 0.5 g twice daily. The duration of treatment was individualized according to symptom improvement. Patients with insomnia were given zopiclone to improve sleep, and active smokers were advised to quit smoking, while passive smokers were encouraged to prohibit smoking in their living spaces.

Outcomes included changes in CAS, degree of exophthalmos, IOP, and extraocular muscle thickness before and after treatment. Specifically, IOP was measured using Goldmann applanation tonometry at baseline and follow‐up visits. Two consecutive readings were obtained and averaged, and all measurements were performed by the same ophthalmologist to minimize interoperator variability. Extraocular muscle thickness was assessed on coronal orbital CT scans (3‐mm slice thickness); the maximal cross‐sectional diameter of each rectus muscle was measured perpendicular to the muscle axis at the point of greatest thickness. Two blinded radiologists independently evaluated the scans, and discrepancies were resolved by consensus. Diplopia was evaluated using both clinical ocular motility examination and the Hess chart. Improvement was defined as disappearance of diplopia in the primary or reading position or a reduction of at least one quadrant in the diplopia field on Hess chart testing. Patient‐reported functional benefit (e.g., improved ability to read or perform daily tasks) was also recorded. The endpoint of therapy was defined as CAS < 1 with stable ocular signs and symptoms for two consecutive visits. Accordingly, the total number of injections was individualized: most patients completed 8–10 injections, whereas a minority with slower improvement required up to 12. Treatment could also be discontinued earlier in cases of significant adverse effects or patient preference.

## 3. Results

### 3.1. General Information

Among the 17 patients included in this study, there were 10 male patients and 7 female patients, with an average age of 47.92 years. Eight patients were active smokers, and four patients were passive smokers (with partners frequently smoking indoors). At the time of TAO diagnosis, 12 patients were diagnosed with concurrent hyperthyroidism, one patient had subclinical hyperthyroidism, one patient had normal thyroid function, and three patients had hypothyroidism. Four patients had undergone radioactive iodine‐131 treatment. The mean TRAb level was 6.89 ± 7.68, with one patient having discontinued intravenous methylprednisolone treatment over 4 months ago. One patient had duration of eye disease of over 1 year and had irregularly used intravenous and oral glucocorticoids and methimazole over 3 months ago. The average degree of exophthalmos was 20.65 ± 2.01 mm. Eight patients experienced diplopia. IOP was within normal range or mildly elevated, with a mean value of 17.48 ± 2.16 mmHg (Table 1).

**Table 1:** Baseline characteristics of enrolled patients.

Patients	Gender	Age (year)	Smoking history	FT3 (pmol/L)	FT4 (pmol/L)	TSH (mIU/L)	TRAb (IU/L)	Treatment regimen
1	Male	36	Yes	4.57	12.1	0.008	1.61	Methimazole
2	Female	40	No	7.72	18.69	0.001	0.77	Methimazole
3	Male	37	Yes	0.59	1.79	85.141	0.88	RAI
4	Male	61	No	3.86	10.01	2.990	2.89	Methimazole
5	Female	37	Yes	5.71	13.76	1.595	12.2	Methimazole
6	Male	41	Yes	17.23	27.05	0.004	7.69	Methimazole
7	Male	39	No	> 30	70.06	0.007	5.12	Methimazole
8	Female	58	Yes	17.05	41.72	0.003	7.86	Methimazole
9	Female	55	No	2.1	3.23	101.775	13.1	RAI
10	Male	72	Yes	3.99	16.61	1.951	2.16	Euthyroidism
11	Male	36	Yes	6.05	18.24	0.078	1.82	Untreated
12	Female	48	Yes	13.72	6.28	3.475	9.67	Methimazole
13	Male	63	Yes	3.65	8.24	0.058	11	Methimazole
14	Male	49	Yes	5.42	13.77	0.125	32	Methimazole
15	Male	48	Yes	6.27	17.15	0.008	2.6	RAI
16	Female	42	No	5.43	19.02	0.003	2.28	RAI
17	Female	29	Yes	6.73	14.86	0.019	3.5	Methimazole

### 3.2. Treatment Outcomes

Following the aforementioned protocol, patients were treated and followed up for 24 weeks. Of the 17 patients, three discontinued after eight injections, 10 after 10 injections, and four after 12 injections. Reasons for early discontinuation included adverse effects attributable to MMF (two patients), patient choice (one patient), and loss to follow‐up (two patients). Six patients experienced improvement in diplopia. Pre‐ and posttreatment assessments revealed significant improvements in extraocular muscle thickness, proptosis, and CAS. Specifically, the average thickness of the lateral rectus muscle decreased (3.24 ± 1.21 vs. 2.75 ± 0.93, *p* < 0.001, Figure 1(a)); the average thickness of the medial rectus muscle decreased (3.78 ± 1.19 vs. 3.43 ± 0.77, *p* = 0.023, Figure 1(b)); the average thickness of the superior rectus muscle decreased (4.58 ± 1.18 vs. 4.07 ± 1.05, *p* = 0.003, Figure 1(c)); and the average thickness of the inferior rectus muscle decreased (4.94 ± 1.97 vs. 4.51 ± 1.86, *p* = 0.024, Figure 1(d)). Concurrently, we assessed changes in proptosis and IOP: following treatment, proptosis significantly decreased (20.65 ± 2.01 vs. 18.23 ± 1.59, *p* < 0.001, Figure 1(e)) and IOP decreased (17.48 ± 2.16 vs. 15.94 ± 1.39, *p* < 0.001, Figure 1(f)). CAS is a crucial indicator used clinically to evaluate the inflammatory activity of eye diseases. Our study found that after 24 weeks of treatment with combined compound betamethasone periocular injection and oral MMF, CASs significantly decreased (4.90 ± 0.83 vs. 0.97 ± 0.91, *p* < 0.001, Figure 1(g)). In this analysis, one eye had a CAS decrease of less than two points, while the remaining eyes had a decrease of two points or more. TRAb, an important pathogenic factor in thyroid‐associated eye diseases, also showed a significant decrease in levels after treatment (6.89 ± 7.68 vs. 2.62 ± 3.04, *p* = 0.01, Figure 1(h)) compared with baseline.

Figure 1The efficacy of combined treatment with superficial orbital injections of compound betamethasone and oral mycophenolate mofetil in active TAO. (a) Lateral rectus muscle comparison; (b) medial rectus muscle comparison; (c) superior rectus muscle comparison; (d) inferior rectus muscle comparison; (e) proptosis comparison; (f) intraocular pressure (IOP) comparison; (g) clinical activity score (CAS) comparison; and (h) TRAb comparison.

**Figure:**
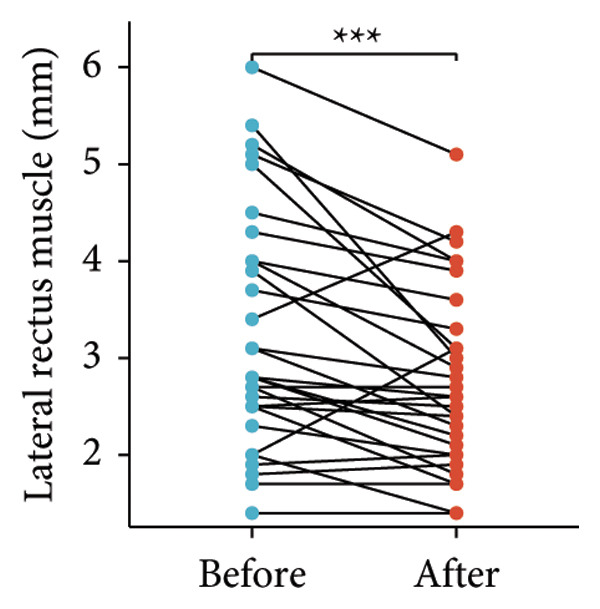
(a)

**Figure:**
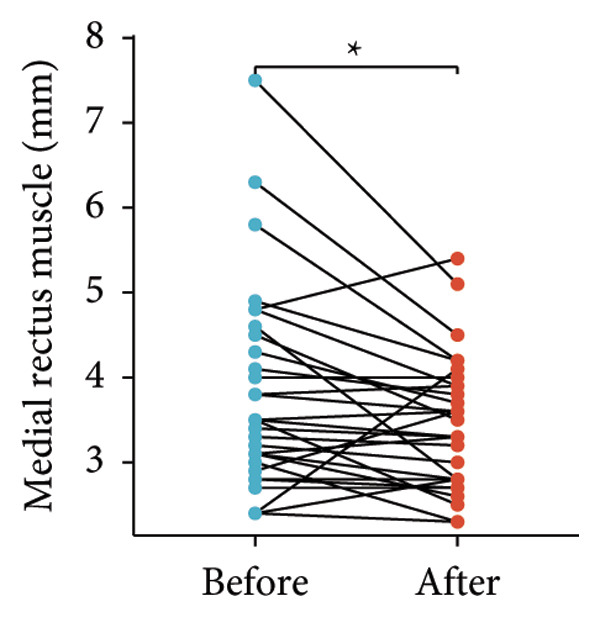
(b)

**Figure:**
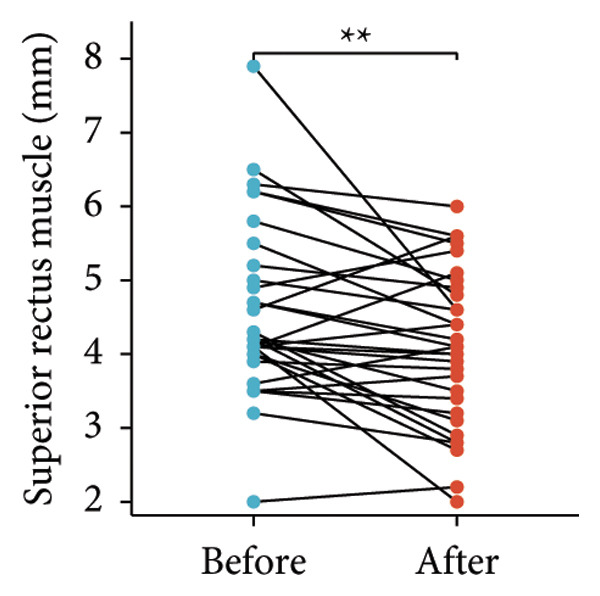
(c)

**Figure:**
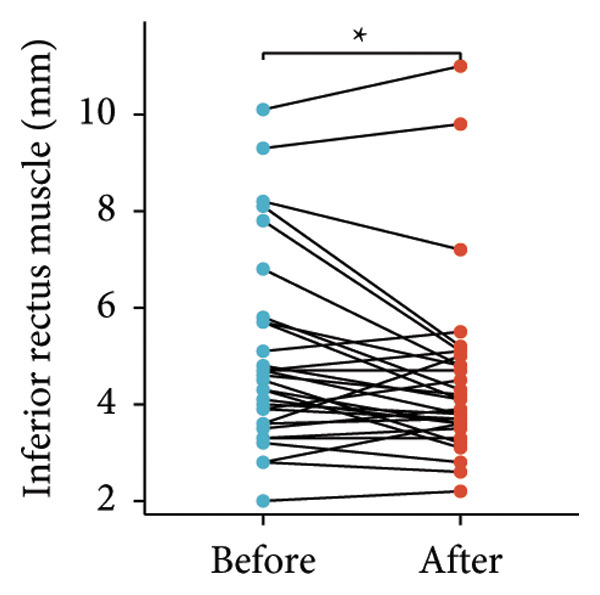
(d)

**Figure:**
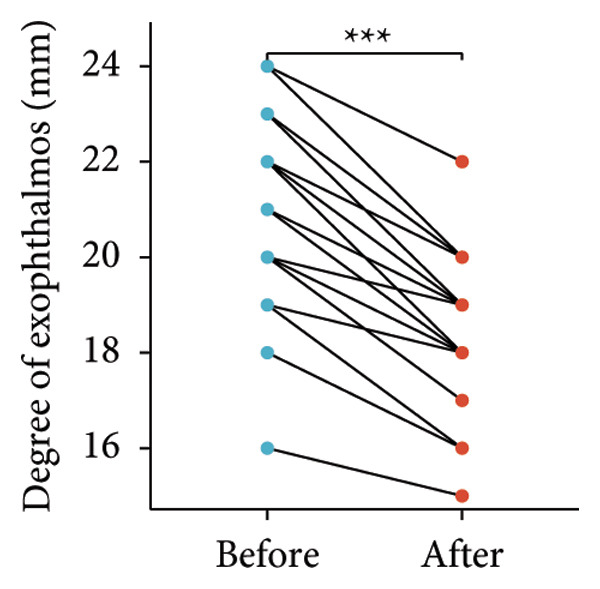
(e)

**Figure:**
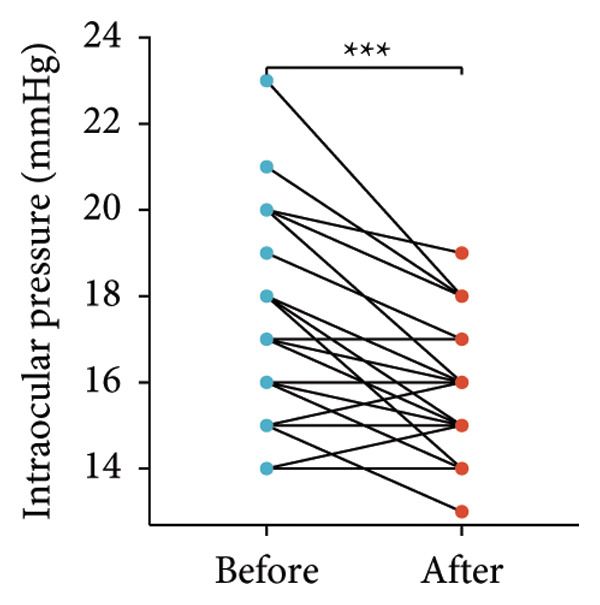
(f)

**Figure:**
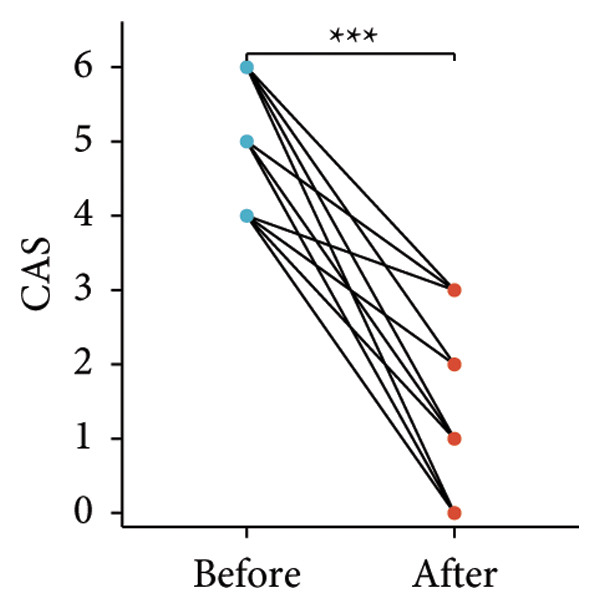
(g)

**Figure:**
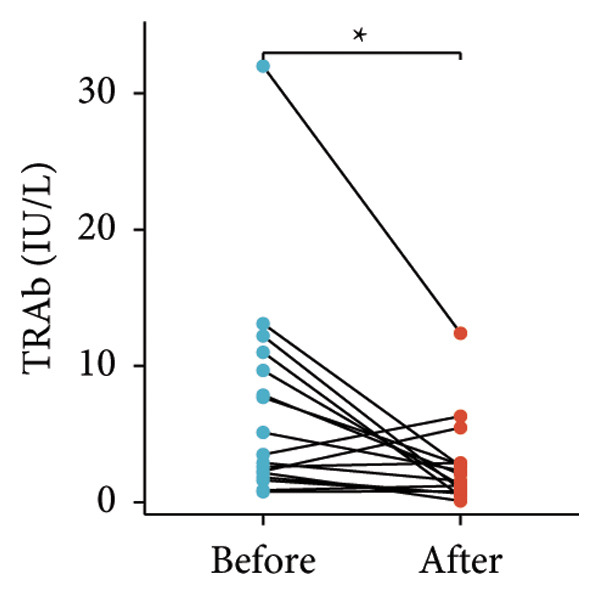
(h)

### 3.3. Influence of Baseline Treatment Regimens on the Efficacy of MMF Combined With Compound Betamethasone Treatment

Radioactive iodine therapy and oral antithyroid drugs are the two main treatment modalities for hyperthyroidism. In this study, 4 patients (7 affected eyes) developed thyroid eye disease after receiving radioactive iodine therapy, while 11 patients (20 affected eyes) were continuously treated with oral methimazole. Subgroup analysis of these two baseline treatment modalities revealed that after 24 weeks of treatment with combined compound betamethasone periocular injection and oral MMF, patients in the RAI group showed a significant decrease in the average thickness of the lateral rectus muscle (Figure 2(a)), while no statistically significant improvements were observed in the medial, superior, and inferior rectus muscles (Figures 2(b), 2(c), and 2(d)); however, proptosis (Figure 2(e)), IOP (Figure 2(f)), and CAS (Figure 2(g)) all significantly improved compared with baseline. Patients in the MMI group showed significant improvement in all these parameters after treatment. Covariance analysis results suggested that the improvement in thyroid‐associated eye disease with combined therapy of compound betamethasone periocular injection and oral MMF is independent of the baseline treatment regimen (RAI or MMI). Given the limited sample size, the subgroup analyses presented in this study should be considered exploratory and hypothesis‐generating rather than confirmatory.

Figure 2Influence of baseline treatment regimens on the efficacy of MMF combined with compound betamethasone treatment. (a) Comparison of lateral rectus muscle; (b) comparison of medial rectus muscle; (c) comparison of superior rectus muscle; (d) comparison of inferior rectus muscle; (e) comparison of proptosis; (f) comparison of intraocular pressure (IOP); and (g) comparison of Clinical Activity Score (CAS).

**Figure:**
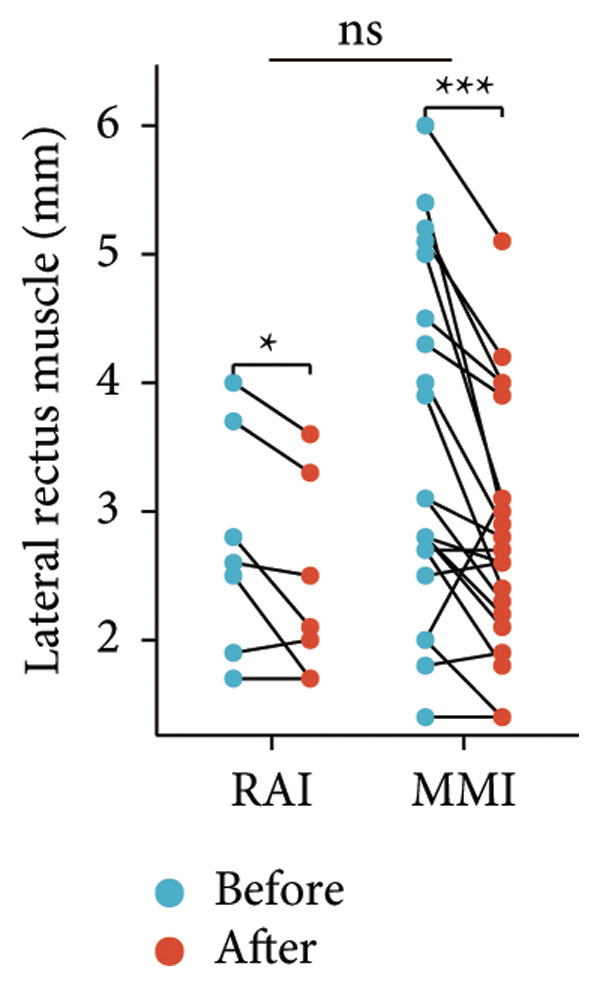
(a)

**Figure:**
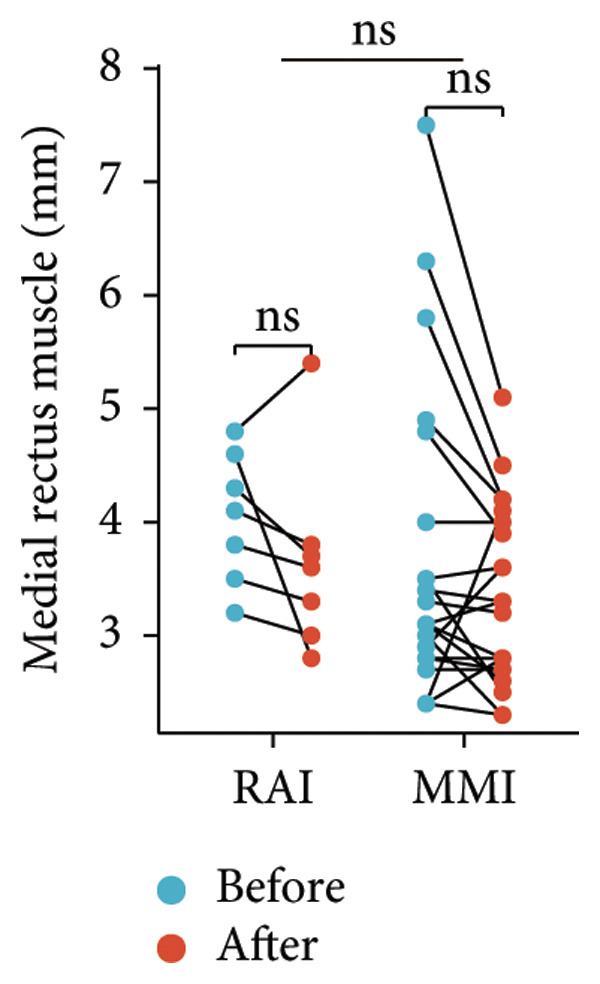
(b)

**Figure:**
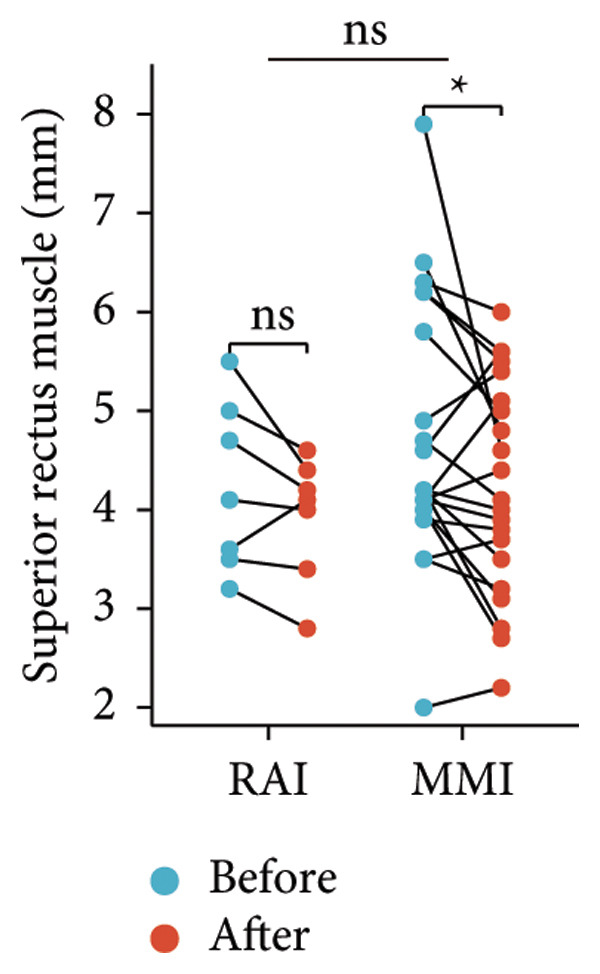
(c)

**Figure:**
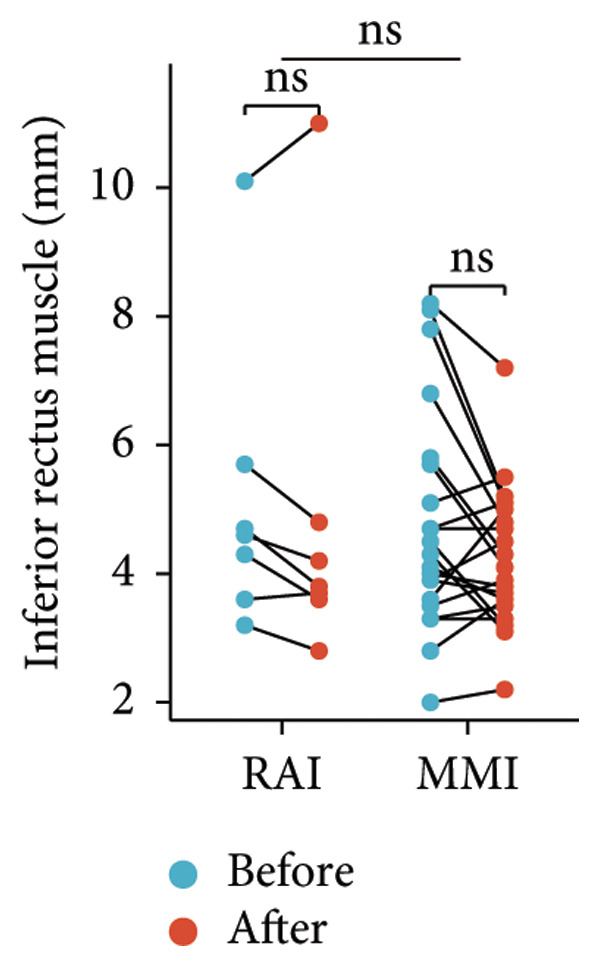
(d)

**Figure:**
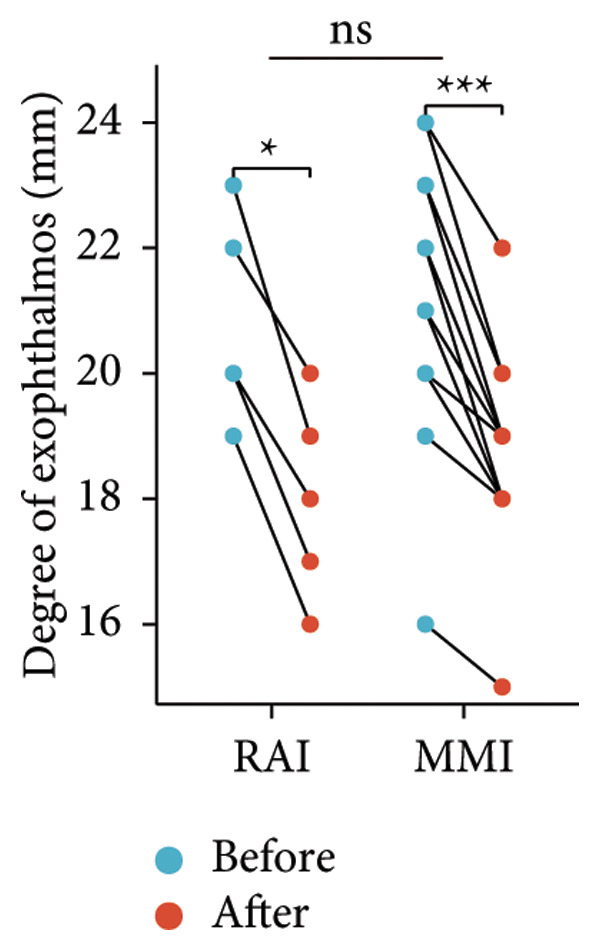
(e)

**Figure:**
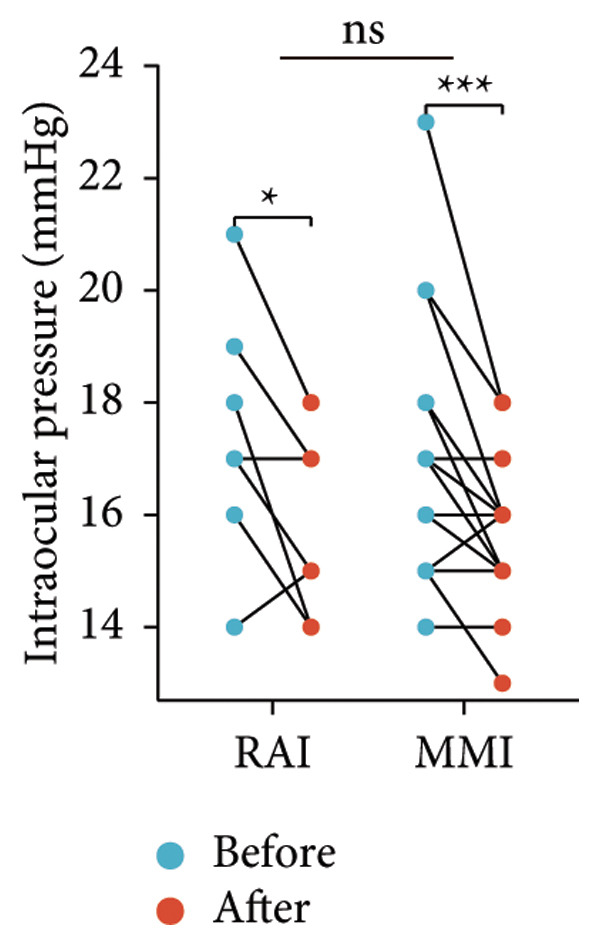
(f)

**Figure:**
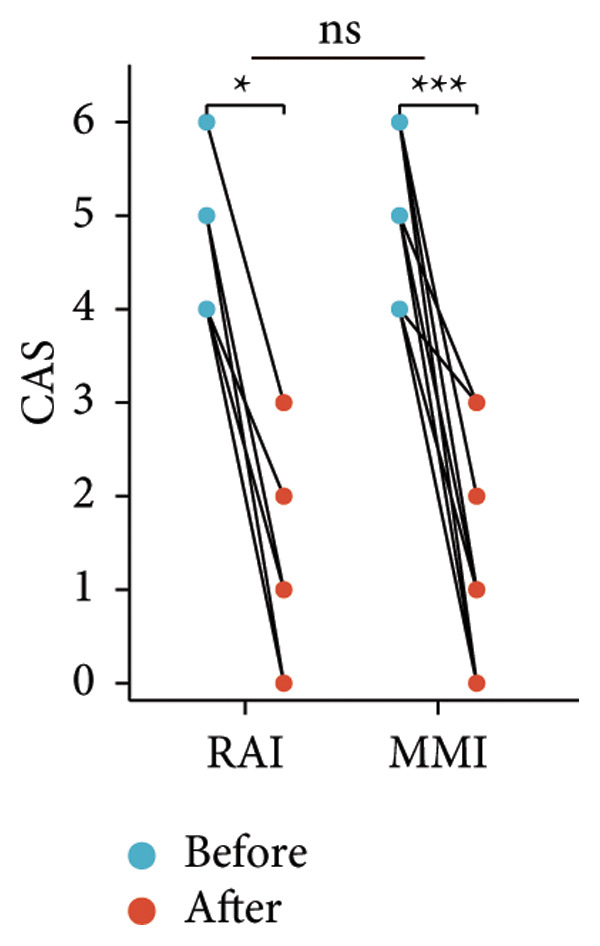
(g)

### 3.4. Analysis of Adverse Effects

One patient experienced impaired glucose tolerance during the treatment period, with a family history of diabetes. However, no other patients developed new‐onset diabetes or impaired glucose tolerance. None of the patients exhibited abnormal elevation of liver transaminases or newly developed hypertension during the course of treatment. One patient reported facial swelling after 2 weeks of MMF use, while another patient complained of generalized fatigue after 1 month of MMF use.

## 4. Discussion

Glucocorticoids remain one of the mainstays in the treatment of TAO. Systemic administration is effective but often limited by adverse effects, leading to growing interest in local steroid delivery. Ebner et al. first reported that retrobulbar compound betamethasone injection improved ocular motility and reduced muscle thickness with minimal side effects. Subsequent studies, both domestic and international, have demonstrated that periocular or periorbital TA injections effectively reduce inflammation and eyelid swelling, although their effects on proptosis and ocular motility remain inconsistent [12–21]. Several comparative studies have shown that periocular steroid injections achieve clinical improvements comparable to oral steroids but with markedly fewer systemic side effects such as weight gain, hyperglycemia, and hypertension [14–21]. Traditionally, TA has been the most widely used agent; however, its strong local irritation, crystallization tendency, and risk of tissue adhesion make it unsuitable for repeated injections, and some studies have reported transient increases in IOP [12, 15–18].

Compound betamethasone, used in our study, contains 2‐mg betamethasone sodium phosphate and 5‐mg betamethasone dipropionate, combining rapid onset with prolonged duration of action. Compared with TA, it causes less local irritation and allows repeated administration. Considering its pharmacokinetic properties (half‐life: 36–54 h and sustained efficacy: 7–11 days), a 2‐week injection interval was deemed appropriate and well tolerated. Most patients experienced symptom relief within several injections, and the total steroid dose was much lower than systemic therapy, minimizing systemic adverse effects. Although our findings suggest favorable outcomes, direct superiority over systemic glucocorticoids cannot be confirmed without randomized controlled trials. Moreover, no significant difference was found between patients who developed TAO after radioactive iodine therapy and those receiving methimazole treatment, suggesting that this combined regimen may be effective regardless of baseline therapy.

MMF exerts immunosuppressive and anti‐inflammatory effects by inhibiting IMPDH in activated B and T lymphocytes, thereby reducing guanosine nucleotide synthesis and suppressing antibody and effector‐cell responses [6, 7]. The main adverse effects of MMF include gastrointestinal discomfort, hematologic abnormalities, and mild hepatic dysfunction, which are generally dose dependent [22, 23]. Previous studies have shown that combining MMF with corticosteroids achieves higher efficacy and lower relapse rates than corticosteroid monotherapy [8–11]. In our study, MMF was well tolerated at low doses; only two patients discontinued due to mild side effects (facial swelling and fatigue). Both improved after continuing local injections alone, but these anecdotal findings are insufficient to assess the efficacy of monotherapy. Although most studies suggest that local steroid therapy is not superior to systemic administration, our results indicate potential benefit in steroid‐refractory cases. One patient with persistent diplopia and CAS = 5 after 4 months of intravenous methylprednisolone improved markedly after switching to periocular compound betamethasone plus MMF, while another long‐term patient also showed symptom relief after adopting this regimen and quitting smoking. None of our patients experienced complications such as IOP elevation, fat atrophy, granuloma, or embolic events previously reported in the literature [24, 25].

In addition to conventional immunosuppressive and corticosteroid‐based regimens, several biologic agents have recently shown promise in TAO. Teprotumumab, an IGF‐1R antagonist, and tocilizumab, an IL‐6 receptor inhibitor, have demonstrated robust efficacy in moderate‐to‐severe or steroid‐resistant disease [26–28]. Rituximab, an anti‐CD20 monoclonal antibody, has shown mixed results across trials [29, 30]. However, the high cost, limited accessibility, and potential systemic risks of biologics restrict their widespread use. In contrast, our combined periocular compound betamethasone plus MMF regimen may represent a more practical and cost‐effective therapeutic option, especially in settings where biologics are unavailable. This study has several limitations. First, the absence of a control arm limits causal inference regarding treatment efficacy. Second, all enrolled patients had normal or mildly elevated IOP, which may reduce the generalizability of the results. Third, although MRI provides superior soft‐tissue resolution, CT remains the standard orbital imaging modality in our center due to accessibility and cost considerations. Finally, the small sample size and relatively short 24‐week follow‐up limit the statistical power of subgroup analyses, which should be regarded as exploratory and hypothesis‐generating only.

In conclusion, periocular compound betamethasone combined with MMF demonstrated promising improvements in inflammatory and structural parameters of TAO. Nevertheless, these results should be interpreted cautiously because of the noncontrolled design, small sample size, and short follow‐up duration. Future large‐scale prospective studies are needed to validate these preliminary findings.

## Ethics Statement

The study protocol was approved by the Ethics Committee of the First Affiliated Hospital of USTC, Division of Life Science and Medicine, University of Science and Technology of China.

## Consent

The authors have nothing to report.

## Disclosure

All authors have reviewed and approved the final version of the manuscript.

## Conflicts of Interest

The authors declare no conflicts of interest.

## Author Contributions

Data acquisition and initial drafting of the work were conducted by Bin Huang, Chunchun Xiao, and Yiwei Liu. The primary statistical analysis was carried out by Bin Huang and Wan Zhou. Interpretation of patient data was performed by Chunchun Xiao and Bin Huang. Shandong Ye substantially revised the manuscript. Chunchun Xiao and Yiwei Liu contributed equally to this work.

## Funding

This study was supported by the Health Commission of Anhui Province Research Project (AHWJ2024BAe20030).

## Data Availability

The datasets analyzed during the current study are available from the corresponding author upon reasonable request.
